# Machine learning based identification of structural brain alterations underlying suicide risk in adolescents

**DOI:** 10.1007/s44192-023-00033-6

**Published:** 2023-02-13

**Authors:** Sahil Bajaj, Karina S. Blair, Matthew Dobbertin, Kaustubh R. Patil, Patrick M. Tyler, Jay L. Ringle, Johannah Bashford-Largo, Avantika Mathur, Jaimie Elowsky, Ahria Dominguez, Lianne Schmaal, R. James R. Blair

**Affiliations:** 1 Multimodal Clinical Neuroimaging Laboratory (MCNL), Center for Neurobehavioral Research, Boys Town National Research Hospital, 14015 Flanagan Blvd. Suite #102, Boys Town, NE USA; 2 Child and Adolescent Psychiatric Inpatient Center, Boys Town National Research Hospital, Boys Town, NE USA; 3 Institute of Neuroscience and Medicine, Brain & Behaviour (INM-7), Research Centre Jülich, Jülich, Germany; 4 Institute of Systems Neuroscience, Medical Faculty, Heinrich Heine University Düsseldorf, Düsseldorf, Germany; 5 Child and Family Translational Research Center, Boys Town National Research Hospital, Boys Town, NE USA; 6 Center for Brain, Biology, and Behavior, University of Nebraska-Lincoln, Lincoln, NE USA; 7 Center for Youth Mental Health, University of Melbourne, Melbourne, VIC Australia; 8 Orygen, Parkville, Australia; 9 Child and Adolescent Mental Health Centre, Mental Health Services, Capital Region of Denmark, Copenhagen, Denmark

**Keywords:** Cortical volume, Morphometry, Brain parcellation, Suicidal ideation, Emotions, Youth

## Abstract

Suicide is the third leading cause of death for individuals between 15 and 19 years of age. The high suicide mortality rate and limited prior success in identifying neuroimaging biomarkers indicate that it is crucial to improve the accuracy of clinical neural signatures underlying suicide risk. The current study implements machine-learning (ML) algorithms to examine structural brain alterations in adolescents that can discriminate individuals with suicide risk from typically developing (TD) adolescents at the individual level. Structural MRI data were collected from 79 adolescents who demonstrated clinical levels of suicide risk and 79 demographically matched TD adolescents. Region-specific cortical/subcortical volume (CV/SCV) was evaluated following whole-brain parcellation into 1000 cortical and 12 subcortical regions. CV/SCV parameters were used as inputs for feature selection and three ML algorithms (i.e., support vector machine [SVM], K-nearest neighbors, and ensemble) to classify adolescents at suicide risk from TD adolescents. The highest classification accuracy of 74.79% (with sensitivity = 75.90%, specificity = 74.07%, and area under the receiver operating characteristic curve = 87.18%) was obtained for CV/SCV data using the SVM classifier. Identified bilateral regions that contributed to the classification mainly included reduced CV within the frontal and temporal cortices but increased volume within the cuneus/precuneus for adolescents at suicide risk relative to TD adolescents. The current data demonstrate an unbiased region-specific ML framework to effectively assess the structural biomarkers of suicide risk. Future studies with larger sample sizes and the inclusion of clinical controls and independent validation data sets are needed to confirm our findings.

**Supplementary Information:**

The online version contains supplementary material available at 10.1007/s44192-023-00033-6.

## Introduction

Suicide is one of the leading causes of death for individuals aged 10–19 years in the United States [1]. The increase in suicide rates has been so dramatic that a recent advisory issued by the USA Surgeon General on Youth Mental Health Crisis underscored the importance of timely data collection and research to determine potential underlying biomarkers [2]. Prior studies indicate that the prediction accuracy of suicide risk is low [3, 4] and relies on self-report/observational measures and *classical statistical approaches* (e.g., group-level comparisons). Potentially, prediction can be improved via identification of functional and structural neural signatures of suicide risk and the use of machine learning (ML) algorithms for statistical analysis. In particular, spatial patterns of structural brain alterations in conjunction with ML can be utilized for diagnostic classification [5]. The current study aims to determine the extent to which identified region-specific cortical and subcortical structural alterations contribute to the classification between adolescents who demonstrate clinically concerning levels of suicide risk and typically developing (TD) adolescents.

Recent work has focused on the neurobiology of suicide risk via a variety of neuroimaging methods, including functional MRI (fMRI) and structural MRI (sMRI) [6]. Previous fMRI studies have suggested that measures of suicide risk (e.g., suicidal ideation and past suicidal attempts [7, 8]) are associated with: (a) several brain regions particularly implicated in emotion processing and mood regulation (e.g., dorsal- and ventral-lateral prefrontal cortices, orbitofrontal cortex, superior frontal gyrus, and anterior cingulate cortex [9–11]); (b) reduced functional hemodynamic response within the left precentral gyrus during a verbal fluency task [12]; and (c) functional abnormalities in regions within the middle and superior temporal cortices [6, 13]. Previous brain morphometry-suicide risk studies are somewhat consistent with fMRI studies and have found some overlapping brain areas with both cortical and subcortical structural alterations in terms of cortical thickness (CT), cortical surface area (CSA), and cortical/subcortical volume (CV/SCV). Cortical thinning/reduced CSA/reduced CV within the left dorsal- and ventral-lateral prefrontal cortices [14, 15], orbitofrontal cortex [15], superior frontal gyrus [16], frontal pole [17], precentral gyrus [18, 19], and anterior cingulate cortex [14] have been associated with high suicide risk. In addition, sMRI work has revealed reduced volume within the middle and superior temporal gyrus in adolescents with a history of suicide attempt relative to comparison adolescents [20–22]. Greater CSA and CV within the dorsolateral prefrontal gyrus have been associated with reduced suicidal ideation [23]. However, other studies have shown opposite effects. For example, compared to depressed suicide non-attempters, depressed suicide attempters have been reported to show either larger CSA or larger CV within the lateral orbitofrontal, postcentral, and lateral occipital areas [16], and compared to non-attempters, suicide attempters showed greater CV of the prefrontal regions [24] and rostral anterior cingulate cortex [25].

In one of the recently published studies on adolescents/young adults diagnosed with major depressive disorder, an ML algorithm was used in conjunction with sMRI data to identify brain structures associated with suicide attempts relative to patients with suicidal ideation but without a history of suicide attempts [26]. In that study, a cross-validation accuracy of 78.59% was reported, and most of the identified regions (among altered CT within the inferior frontal cortex, anterior cingulate cortex, posterior cingulate cortex, and fusiform gyrus and altered CV within the anterior cingulate cortex and temporal pole) had a significant overlap with prior fMRI and sMRI work on suicide risk.

Summarizing based on the literature, we can conclude that, *first*, prior work has identified *widespread* cortical alterations (with inconsistent directionality) that were associated with suicide risk. *Second*, prior sMRI work on suicide risk in adolescents has mostly used traditional data analysis techniques and, to a greater extent, has lacked the evaluation of ML approaches. Therefore, prior work does not allow making predictions at the individual level, which is critical for the clinical translation of identified biomarkers. Thus, the goal of the current study was to address previous challenges by using multiple ML classifiers to distinguish adolescents at suicide risk and TD adolescents and to further determine which of the ML classifiers leads to the most accurate group differentiation. The current study specifically focused on volumetric measures, as this measure combines both cortical thickness and cortical surface area information. In other words, thickness and surface area measurements influence volumetric measurements [27, 28]. Though thickness and surface area individually may improve the specificity compared to volume, the joint analysis of thickness and surface area in terms of volume may be potentially more informative to simultaneously quantify the effects of thickness and surface area [29].

The current study of adolescents at suicide risk and TD adolescents: (i) used a fine whole-brain parcellation (including 1000 cortical [30] and 12 subcortical regions [31]); (ii) used CV/SCV measures; and (iii) assessed the performance of three ML models (i.e., support vector machine [SVM] [32], k-nearest neighbors [k-NN] [33], and ensemble [ENS] [34]). Based on prior published/cited work on suicide risk, we predicted that the structural brain alterations within the dorsal- and ventral-lateral prefrontal cortices, orbitofrontal/inferior frontal cortex, superior frontal gyrus, precentral gyrus, cingulate cortex, and superior and middle temporal cortices would contribute to the classification between adolescents at suicide risk and TD adolescents.

## Methods

### Participants

The current study included data collected from 79 adolescents recruited from a residential care facility (age range = 13–19 years, mean age = 16.26 ± 1.18 years, 36 females, IQ range = 76–133, and mean IQ = 99.62 ± 13.60) who demonstrated clinically concerning levels of suicide risk (Suicide Probability Scale [SPS] range = 60 T-77 T, mean SPS = 66.18 ± 4.67) and 79 typically developing (TD) adolescents recruited from the surrounding community (age range = 13–19 years, mean age = 15.94 ± 1.48 years, 26 females, IQ range = 79–119, and mean IQ = 103.11 ± 9.00) (see Table 1). An SPS score of more than 59 T reflects clinically concerning levels of suicide risk [35]. For more details, please see Supplementary Section S1.

**Table 1:** Demographic characteristics and group differences

Characteristics	Adolescents at suicide risk (N = 79)	TD adolescents (N = 79)	Difference/correlations with total SPS scores
Sex (F/M)	36/43	26/53	*p* = 0.13 (Chi squared)
Mean Age (SD)	16.26 (1.18)	15.94 (1.48)	*p* = 0.13 (two-sample *t-test*)
Mean IQ (SD)	99.62 (13.60)	103.11 (9.00)	*p* = 0.06 (two-sample *t-test*)
*Measures of suicide risk*			
Mean SUI (SD)	61.37 (8.91)	–	–
Mean HOP (SD)	64.92 (5.41)	–	–
Mean NEG (SD)	60.05 (8.56)	–	–
Mean HOST (SD)	62.16 (6.82)	–	–
Mean total SPS scores (SD)	66.18 (4.67)	–	–
*Comorbidities/diagnoses (N)*			
ADHD (%)	60 (75.95)	–	− 0.04
CD (%)	48 (60.76)	–	− 0.30**
MDD (%)	20 (25.32)	–	0.30**
PTSD (%)	17 (21.52)	–	0.18
GAD (%)	31 (39.24)	–	0.29**
SAD (%)	27 (34.18)	–	0.14
*Medications (N)*			
Antipsychotic (%)	9 (11.39)	–	− 0.07
SSRIs (%)	20 (25.32)	–	0.16
Stimulants (%)	18 (22.78)	–	− 0.10

TD, typically developing; SPS, suicide probability scale; F/M, female/male; SD, standard deviation; IQ, intelligence quotient; SUI, suicidal ideation; HOP, hopelessness; NEG, negative self-evaluations; HOST, hostility; ADHD, attention-deficit/hyperactivity disorder; CD, conduct disorder; MDD, major depressive disorder; PTSD, post-traumatic stress disorder; GAD, generalized anxiety disorder; SAD, social anxiety disorder; SSRIs, selective serotonin reuptake inhibitors

*p < 0.05

**p < 0.01

All the included participants and their parents provided written informed assent/consent prior to enrollment. The study protocol was approved by the Institutional Review Board at Boys Town National Research Hospital (BTNRH). All procedures performed in this study were conducted in accordance with the ethical standards of the institutional and/or national research committee and with the 1964 Helsinki declaration and its later amendments or comparable ethical standards.

### Data collection

#### Neuroanatomical data

High-resolution sMRI data were collected using a 3 T Siemens MRI scanner located at BTNRH. Each participant was instructed to try their best to minimize head movement during the entire scan. Whole‐brain anatomical data for each participant were acquired using a 3D magnetization‐prepared rapid acquisition gradient echo (MPRAGE) sequence, which consisted of 176 axial slices (matrix size = 256 × 208; slice thickness = 1 mm, voxel resolution = 0.9 × 0.9 × 1 mm^3^, field of view (FOV) = 230 mm, flip angle = 8^°^, repetition time = 2200 ms, echo time = 2.48 ms).

#### General intelligence (IQ)

The Full-Scale IQ-2 Subtests (FSIQ-2) from FSIQ-4 (WASI-II) [36] were used to estimate IQ in the domains of vocabulary and matrix reasoning. The FSIQ-2 scores have a high reliability coefficient (*α* = 0.93) in children aged between 6 and 16 years [37] and a strong correlation of (*r* = 0.94) with FSIQ-4 [38].

#### Suicide probability scale (SPS)

The SPS is a 36-item self-report measure of global suicide risk in adolescents and adults [35]. The scale assesses the severity of four symptoms—suicidal, hopelessness, negative self-evaluations, and hostility [35]. Prior work has shown that the SPS is a valid and reliable measure of suicide risk with high internal consistency (Cronbach’s alpha = 0.91) [35, 39, 40].

### Data analysis

#### Image preprocessing

The *recon‐all* pipeline from the FreeSurfer toolbox (Version 6.0; https://surfer.nmr.mgh.harvard.edu) was used to process the anatomical brain images [41, 42]. Processing of anatomical images involved basic image preprocessing steps, including head motion correction, removal of non-brain tissue (i.e., brain extraction), automated transformation to the standard template space, volumetric segmentation into cortical and subcortical matter, intensity correction, and parcellation of the cerebral cortex into gyral and sulcal matter [43]. The technical details of these steps can be found in previous publications [41, 42, 44]. To inspect the preprocessing accuracy, standard quality control steps, including a careful visual inspection of raw structural images, skull‐stripped brain volumes, pial surfaces (both lateral and mid-sagittal views), and internal and external surface segmentations (to determine the accuracy of cortical thickness and cortical surface segmentations), were performed. None of the participants failed the quality inspection criteria, and therefore, none of the participants were excluded from further analysis.

#### Estimation of region-specific morphometry measures

**Fig. 1:**
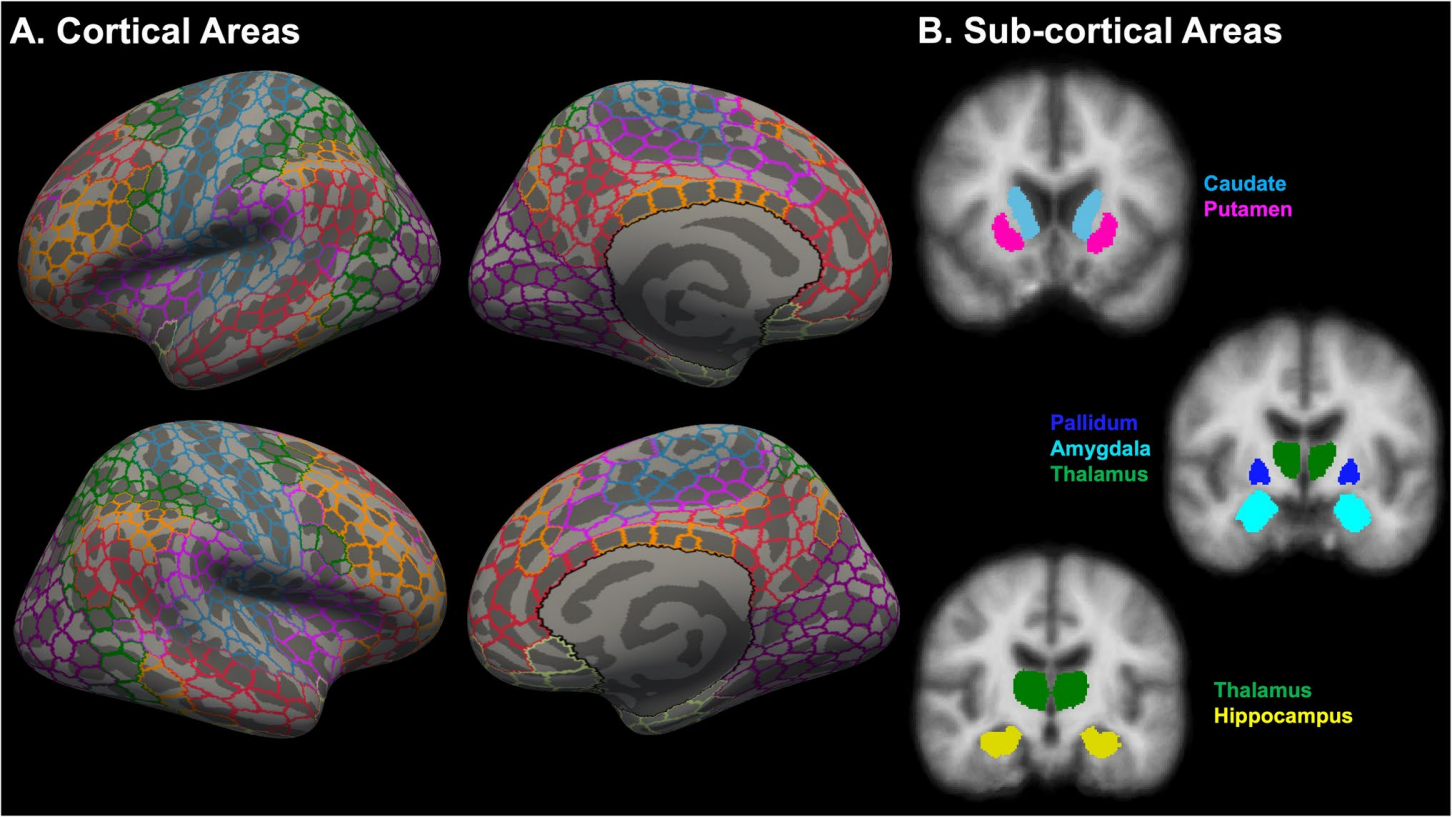
Whole-brain parcellation into 1000 cortical and 12 subcortical regions. Schaefer’s atlas (**A**) and whole-brain default automated segmentation (**B**) were used to parcellate the whole brain into 1000 cortical (i.e., 500 cortical regions per hemisphere) and 12 subcortical regions (i.e., 6 subcortical regions per hemisphere), respectively

Schaefer’s atlas [30] and whole-brain default automated segmentation [31] from FreeSurfer were used to parcellate the whole brain into 1000 cortical (i.e., 500 regions per hemisphere) (Fig. 1A) and 12 subcortical (i.e., 6 regions per hemisphere) (Fig. 1B) regions. Subjectwise measures of CV for bilateral cortical areas, SCV for bilateral subcortical areas, and intracranial volume (ICV; a measure of head size) were evaluated using the *mri_surf2surf*, *mris_anatomical_stats*, and *aparcstats2table* pipelines following the FreeSurfer *recon‐all* pipeline.

#### Data preparation

Estimation of CV values of 1000 cortical regions and SCV of 12 subcortical regions for each participant resulted in a total of 1012 features corresponding to volumetric measures (i.e., CV and SCV) for each subject. CV/SCV values were residualized w.r.t. age, sex, IQ, and head size. MATLAB R2022a was used to estimate the residualized values of CV/SCV.

#### Feature identification and ML analysis

**Fig. 2:**
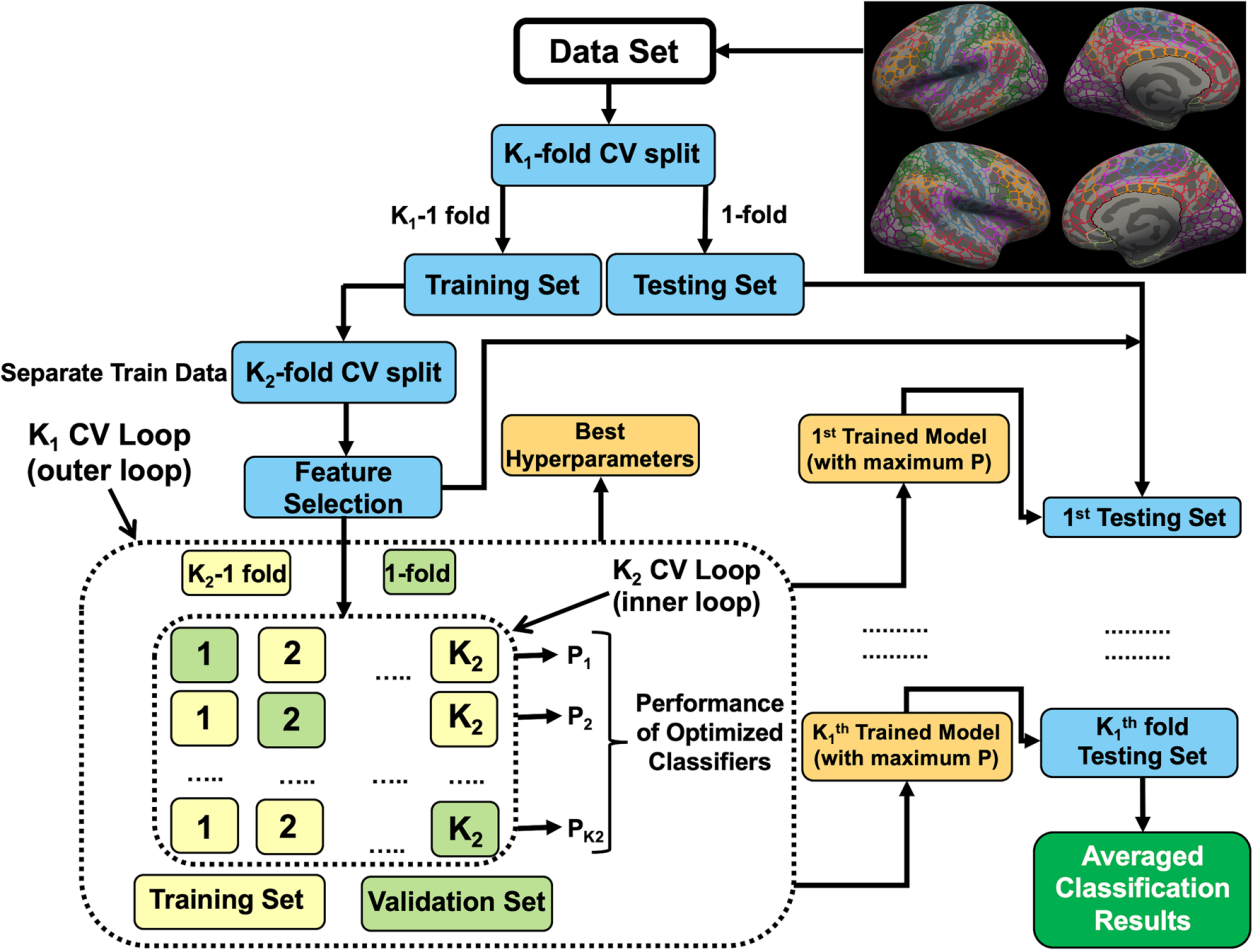
Machine learning (ML) framework. Here, we describe an overview of the ML framework used in conjunction with feature identification, K_1_ × K_2_ nested cross-validation, and whole-brain morphometry data (i.e., cortical volume [CV]) to classify adolescents at suicide risk and typically developing (TD) adolescents

Feature selection and ML analysis were performed in MATLAB R2022a. We used a K_1_ x K_2_ (here, K_1_ = 10 and K_2_ = 10) nested cross-validation approach [45, 46], where K_1_ and K_2_ represent the number of outer and inner loops, respectively. The CV/SCV set was randomized. First, the data were split into K_1_ outer folds (outer loop). Within each iteration of the outer fold, K_1_-1 folds were used as training data sets, and the remaining fold was used as the testing data set. Within the outer loop, the K_1_-1 folds of the training data set features were transformed into z-score, and corresponding transformations were applied to the testing data set. In the inner loop, the training data set was divided into K_2_ folds (inner loop), where K_2_-1 folds were used as the subtraining data set, and the remaining fold was used as the validation data set. Least absolute shrinkage and selection operator (LASSO) feature selection [47, 48] (function *lasso* from MATLAB R2022a) was applied to the subtraining data set. The feature selection procedure was repeated K_2_ times by alternating the subtraining and validation sets. This process resulted in K_2_ sets of best features. The final set of features comprised features that appeared at least 50% times in the K_2_ sets of best features. The idea of using 50% consensus features was inspired by previous work where researchers used a 100% consensus nested cross-validation approach [49]. Three different classification algorithms, namely, SVM, k-NN, and ENS (briefly discussed in Supplementary section S2), were trained on the subtraining data set only using the final features selected and then validated using the validation data set. The selection of the feature selection method LASSO and three algorithms was based on the scikit-learn algorithm flowchart [50]. During parameter tuning, the default settings (within *fitcsvm, fitcknn, and fitcens* implemented in MATLAB R2022a) of the Bayesian Optimization were used. The process was repeated K_2_ times, resulting in K_2_ number of models and their validation accuracies. The model that gave the highest validation accuracy was then tested on the test data set from the outer loop. This process was repeated K_1_ times for each of the three classification algorithms. The performance parameters (i.e., accuracy, sensitivity, and specificity) corresponding to each outer test fold were averaged to find the generalized accuracy (ACC), generalized sensitivity (SEN), and generalized specificity (SPEC) for each classification algorithm. In addition, the area under the receiver operating characteristic curve (AUC) was also used to assess model performance. The *rocmetrics* from MATLAB R2022a was used to estimate the AUC. The final reported set of features represents the aggregated features over K_1_ outer iterations where each outer iteration corresponds to one set of features that appeared at least 50% times in the K_2_ sets of best features. In Fig. 2, we describe our overall ML framework.

#### Follow-up analyses

##### Potential confounds: impact of other major psychopathologies and prescribed medications

A number of our participants were diagnosed with different psychiatric disorders including Attention Deficit Hyperactivity Disorder (*N* = 60), Conduct Disorder (*N* = 48), Major Depressive Disorder (*N* = 20), Post-Traumatic Stress Disorder (*N* = 17), Generalized Anxiety Disorder (*N* = 31), and Social Anxiety Disorder (*N* = 27). In addition, several of our youth were on psychiatric medications (*N* = 47) during the time of the study, including SSRIs, stimulants, and antipsychotics. Table 1 shows demographic characteristics of both comorbidities and medications. Given the potential confounds, the feature identification and ML analysis described above was repeated for those comorbidities/diagnoses (with vs. without) and medications (with vs. without) that showed positive significant associations with SPS scores.

## Results

### Feature identification

**Fig. 3:**
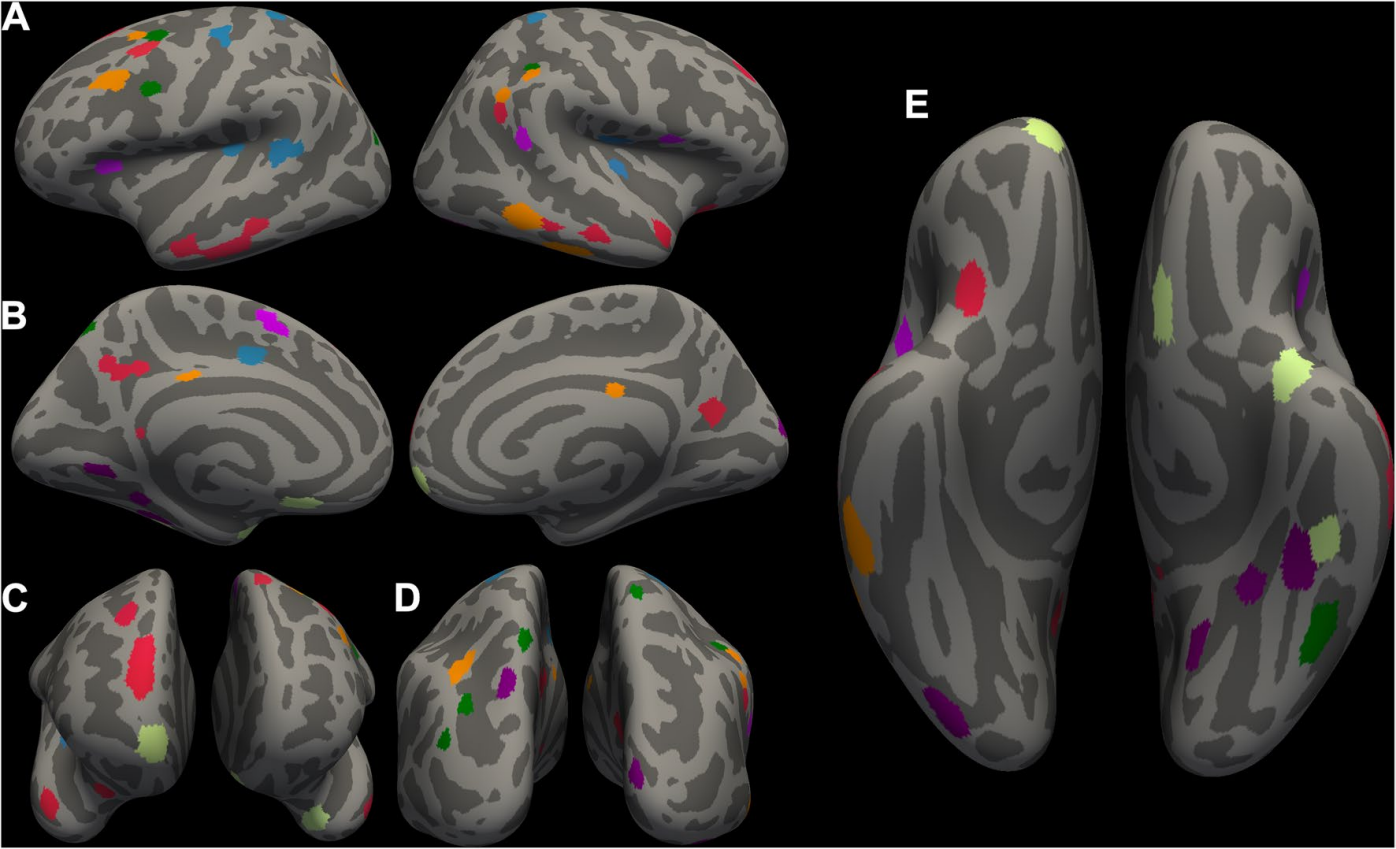
Identified features/regions that contributed to classification accuracy. A total of 62 features for CV/SCV were identified that contributed to the performance parameters

**Fig. 4:**
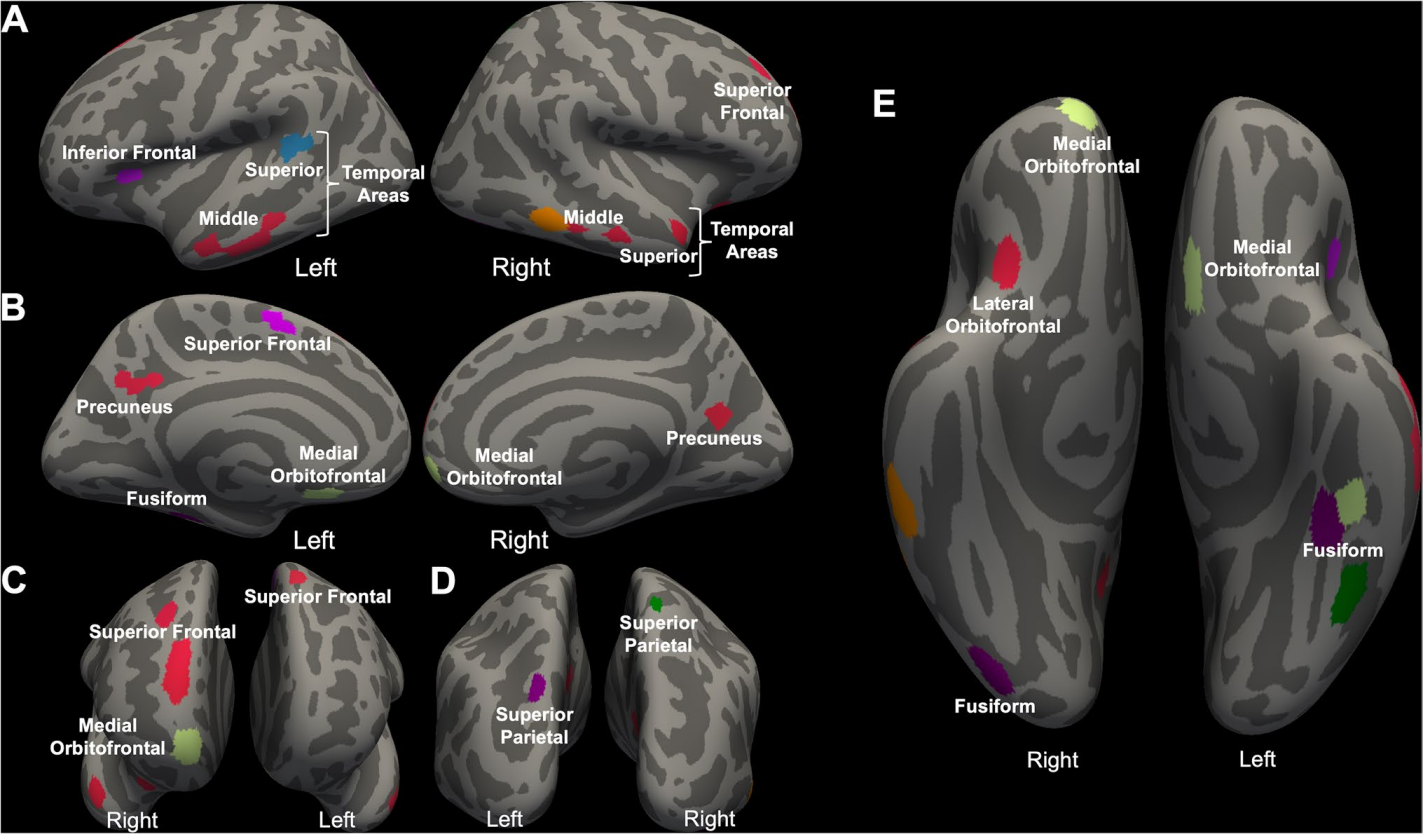
Identified features/regions that contributed bilaterally to classification accuracy. Here, we showed only the features that bilaterally contributed to the performance parameters (**A**–**E**)

A total of 62 features were robustly identified through CV/SCV features; see Table 2 for a detailed list of regions (both bilateral and unilateral) constituting these features. The identified regions that were bilateral mainly included: (a) reduced CV within the frontal and temporal cortices; and (b) increased CV within the precuneus and superior parietal cortex. In Fig. 3, we show the anatomical locations of all the regions constituting 62 identified features (A–E). In Fig. 4, we show only the features that contributed bilaterally (A–E).

**Table 2:** Laterality, morphometry measures, direction, and regions (number of features) associated with machine learning performance measures

Bilateral	Left hemisphere	Right hemisphere
*Cortical volume (CV): adolescents at suicide risk* < *TD*		
Superior frontal gyrus (5)	Caudal middle frontal gyrus (4)	Inferior temporal cortex (1)
Inferior frontal gyrus (2)	Temporal pole (1)	Insula (1)
Orbitofrontal cortex (3)	Pre/post-central gyrus (3)	
Superior temporal gyrus (3)	Inferior parietal cortex (4)	
Middle temporal gyrus (6)	Superior parietal cortex (1)	
Fusiform gyrus (4)	Posterior cingulate cortex (2)	
	Isthmus cingulate cortex (1)	
	Thalamus (1)	
	Lingual gyrus (1)	
*Cortical volume (CV): adolescents at suicide risk* > *TD*		
Precuneus (4)	Superior frontal gyrus (2)	Cuneus (1)
Superior parietal cortex (2)	Transverse temporal gyrus (1)	Post-central gyrus (2)
	Parahippocampal gyrus (1)	Supramarginal gyrus (3)
		Angular gyrus (2)
		Superior corpus callosum (1)

TD, typically developing

### Generalized model performance

The SVM yielded the best model performance measures. The ACC, SEN, SPEC, and AUC were 74.79%, 75.90%, 74.07%, and 87.18%, *respectively*. The k-NN yielded ACC, SEN, SPEC, and AUC values of 73.12%, 78.72%, 68.03%, and 81.42%, *respectively*. The ENS yielded ACC, SEN, SPEC, and AUC values of 63.54%, 66.68%, 62.47%, and 80.28%, *respectively*.

### Impact of other major psychopathologies and prescribed medications

There was significant positive association between: (a) MDD diagnoses and SPS scores (*r* = 0.30, *p* < 0.01); and (b) GAD diagnoses and SPS scores (*r* = 0.29, *p* < 0.01) (Table 1). Because our sample sizes for depressed (N = 20) vs non-depressed (N = 138) and anxious (N = 31) vs non-anxious (N = 127) groups were unbalanced, therefore, we calculated *F-*measure to evaluate the performance of our SVM model. Our follow-up ML analysis yielded *F-*measures of 59.39% and 63.45% to classify depressed and non-depressed adolescents and anxious vs non-anxious adolescents respectively.

## Discussion

The current study implemented a whole-brain 1012-area parcellation approach in conjunction with a sophisticated ML approach to identify structurally altered brain regions that contribute to the classification between adolescents who were at clinically concerning levels of suicide risk and TD adolescents. The identified bilateral regions that contributed to performance parameters mainly included reduced CV within the superior frontal gyrus, regions within the inferior frontal gyrus, orbitofrontal cortex, regions within the superior and middle temporal gyrus, and fusiform gyrus, and increased CV within the precuneus and superior parietal cortex. Finally, we concluded that the SVM was the best performing ML algorithm (relative to k-NN and ENS) in detecting structural biomarkers underlying suicide risk.

Consistent with our a priori predictions, the identified alterations in the current study, particularly within the components of the frontal and temporal cortices [19, 51], appear to be the most consistent with previous functional and structural neuroimaging studies. The components within these cortices included reduced CT, reduced CSA, and/or reduced CV within the superior frontal gyrus [16, 52], inferior frontal gyrus [15, 19, 53, 54], orbitofrontal cortex [55], superior temporal [18, 20, 21, 52, 53], middle temporal gyri [22], and fusiform gyrus [56, 57].

The frontal lobe is functionally involved in planning and rationalizing emotional behavior. The abnormalities within the frontal lobe (particularly specific portions of the superior frontal gyrus and regions within the inferior frontal gyrus) may limit inhibition of the dysregulated emotional limbic system [15, 17, 19, 58, 59]. More specifically, the superior frontal gyrus is associated with inattentional impulsivity [59] and impulsive responses in individuals with posttraumatic stress disorder [60], and the inferior frontal gyrus is known to interpret stimuli in the environment (e.g., inhibiting environment stressors that may cause suicidal behavior) [15]. The orbitofrontal cortex is one of the key components that regulates emotions and impulse, and therefore, structural abnormalities within this region can potentially increase suicide risk [61].

Poor interaction between frontal and limbic systems is implicated in impaired cognitive control and poor impulse control, which are core characteristics of suicidal individuals [62]. Regions within the superior temporal gyrus are implicated in regulating attention to emotions [63], social emotional processing [64], and severity of auditory hallucinations, which could modulate the characteristics associated with suicide risk [63]. Regions within the middle temporal gyrus are implicated while viewing negative (versus positive) facial expressions during an emotion perception task [65]. In a meta-analysis study conducted by Li and colleagues, it was found that brain activation in suicide attempters decreased in the bilateral fusiform gyrus compared to non-attempters across multiple learning-based fMRI tasks [66]. In particular, the fusiform gyrus is involved in facial recognition and perceiving emotions in facial stimuli [67]. Ai and colleagues reported that participants with past suicide attempts had lower activation within the fusiform gyrus during emotional face processing [68].

Our findings also support the involvement of brain regions that are located beyond the frontal and temporal lobes (i.e., increased CV within the bilateral precuneus and superior parietal cortex for adolescents at suicide risk relative to TD adolescents). The precuneus represents the posteromedial portion of the parietal lobe. The abnormalities in amygdala-precuneus/cuneus resting-state functional connectivity are associated with suicidal ideation in female participants with first-episode MDD relative to female participants with first-episode MDD without suicidal ideation and those in the healthy control group [69]. The precuneus supports the internal mental representation of the self and is involved in internally guided attention and manipulation of mental images [70]. Both negative self-representation/self-stigma and attentional bias towards negative stimulus have been well known to be associated with suicidal behavior [71–73]. Our findings are further consistent with prior work in which a larger CV within the superior parietal lobe was found in suicidal patients [22]. Regions within the parietal lobule are involved in organization, decision-making, evaluating outcomes for uncertain future response choices, and cognitive and emotional processing [74]. The functional connections between the prefrontal regions and cortical structures within the parietal lobe (particularly the precuneus) and temporal regions (i.e., part of the default-mode network [DMN]) are associated with self-referential processing, social cognition, and prospective imagination [6]. The altered functional connectivity within the DMN underlines excessive rumination in patients with depression and, hence, the pathophysiology of suicidal risk [75, 76].

It should be noted that we observed opposite effects (i.e., CV decreases within the frontal and temporal regions and increases in the parietal regions). As explained previously, though our findings related to CV decreases within the frontal and temporal regions are in accordance with our a priori predictions and appear to be the most consistent with previous neuroimaging studies. However, the opposite effects found in the parietal regions was surprising. We would argue that both increased and decreased CV may indicate a deviation from typical developmental trajectories of the population under study. Second, since our sample size was not large and, therefore, several biases (e.g., a type II error) cannot be ruled out. In addition, our suicide group showed several psychiatric comorbidities, and some of the participants were also receiving medications. This could also have confounded our results pertaining to opposite effects.

Our study identified SVM as the best trained classifier to detect suicide risk with a reliable classification accuracy in the test sample. SVM is one of the most consistently employed classifiers in prior clinical neuroimaging studies involving schizophrenia, autism spectrum disorder, psychosis [77], MDD [78], Alzheimer’s disease [79], suicidal behavior [26], and other clinically relevant neurological phenotypes [80]. The strengths that make SVM one of the most reliable and extremely popular classifiers in neuroimaging include its ability to yield competitive predictive performance despite having smaller sample sizes, lower risk of overfitting despite having high-dimensional imaging data, classification of subtle brain differences due to its multivariate nature, and flexibility for both linear and nonlinear discriminatory analyses [77, 81–83]. It also has the ability to make inferences at the individual level [a characteristic that is extremely helpful when classifying psychiatric patients (such as suicidal individuals) having within-group heterogeneity] [84].

There are three main caveats to the study that are worth mentioning. First, the sample sizes of both groups (i.e., adolescents at suicide risk and TD adolescents) were relatively small. The current study did not include clinical controls (i.e., individuals with mental disorders without any suicide risk as a comparison group). Therefore, the current sample may have missed identifying some regions that are still relevant to suicide risk. A replication with a larger sample size and inclusion of a third group of clinical controls would be beneficial in future studies. Second, identified regions of interest were interpreted only if they contributed bilaterally to the performance parameters. Therefore, hemispheric laterality was not considered when interpreting the findings. However, to mitigate this concern, region-specific details, along with laterality and the number of features contributing to that region, are summarized in Table 2. Third, the participants at suicide risk showed psychiatric comorbidities (including depression and anxiety), and some of those participants were receiving medications. Therefore, psychiatric comorbidities and psychiatric medications may have confounded the performance parameters. In other words, it’s very challenging to know whether any results obtained are due to suicide risk or may be due to depression or anxiety symptoms. However, to mitigate this concern, our follow-up analysis showed that there is minimal (if any) influence of depression and anxiety on our findings about suicide risk. Other measures of diagnoses and medication status were not associated with severity of suicide risk.

Some of the unique merits of the current study include careful selection of the study sample and utilization of ML algorithms. First, the study sample included adolescents who were at clinically concerning levels of suicide risk and had an age range between 13 and 19 years, an understudied age range particularly associated with heightened suicide risk in youth. Second, to our knowledge, prior neuroimaging studies that used highly sophisticated frameworks in conjunction with multiple ML algorithms to study suicide risk in adolescents are extremely rare [26]. Previous studies mostly used a *variety* of classical statistical approaches and advanced our basic knowledge about brain markers underlying suicide risk. However, our current study identified a specific classifier (i.e., SVM) to detect brain structures underlying suicide risk at the individual-level. As discussed before, most of the brain regions identified that contributed to the classification accuracy highly converge with the previous findings that emerged from completely different statistical approaches—but now findings are more robust and independent of statistical constraints and assumptions, and identified patterns are spatially more expanded. We believe that if the identified biomarkers are reproducible at the patient level, then these biomarkers can be further used as treatment targets, allowing intervention efficacy to improve dramatically.

## Supplementary Information

Below is the link to the electronic supplementary material.

Supplementary file1 (DOCX 24 KB)

## Data Availability

The data that support the findings of this study are available from the corresponding author upon reasonable request. The data are not publicly available due to IRB restrictions.
