# Supplementary material for: Machine learning based identification of structural brain alterations underlying suicide risk in adolescents
Source: Discov Ment Health. 2023 Feb 13;3(1):6. doi: 10.1007/s44192-023-00033-6 (PMC10501026; doi:10.1007/s44192-023-00033-6)
Supplement: Supplementary file 1 — Supplementary file1 (DOCX 24 KB) [file 44192_2023_33_MOESM1_ESM.docx]

**Supplementary Section S1:**

Initially, a total of 186 adolescents (age range = 12-19 years, 68 females, and SPS range 33T-77T) were clinically categorized into the suicide risk category using the Child Suicide Risk Assessment (CSRA) through psychiatric interviews [1], and 178 adolescents (age range = 10-19 years, IQ range = 79-142, and 67 females) were categorized into the TD category. However, for the current study, a total of 79 adolescents were sub-categorized into a stringent suicide risk category by having a total SPS score of 60T or above. A cut-off SPS threshold of more than 59T has been suggested to indicate the need for a careful clinical evaluation of suicide risk [2]. This led to final clinical data set of 79 adolescents (36 females and 43 males with age and IQ range of 13-19 years and 76-133 respectively), who demonstrated clinically concerning levels of suicide risk (SPS range = 60T-77T). As a comparison group, a total of 105 adolescents in the identical age and IQ range of 13-19 years and 76-133, respectively, were categorized in TD healthy-control category. Our initial analysis showed that there were significant group differences in age [*F*(1,183) = 9.55, *p =* 0.002] and IQ [*F*(1,183) = 14.93, *p <* 0.001]. Therefore, prior to conducting any further analysis, participants were removed one-by-one from the TD category in such a way to minimize the differences (to reach non-significant levels) in age and IQ and simultaneously keeping the sex differences as non-significant. This led to a TD data set of 79 adolescents (26 females and 53 males with an age and IQ range of 13-19 years and 79-119 respectively). Our final analysis on revised clinical and TD datasets (N = 79 each) showed that there were no group differences in sex (Chi-squared: *χ^2^* = 2.65, *p =* 0.10), age (two-sample *t*-test: *t*(156) = -1.50, *p* = 0.13), and IQ (two sample *t*-test: *t*(156) = 1.90, *p* = 0.06) (see Table 1). All the above analysis were performed in SPSS 25.

Participants recruited from the residential facility had been referred for behavioral and mental health problems, whereas participants from the community were recruited through flyers or social media. For both sets of participants, inclusion criteria included IQ > 75 and exclusion criteria included pregnancy, claustrophobia, presence of metallic objects in the body (e.g., pacemakers, metal plates, etc.), non-psychiatric medical conditions that require the use of medication that may have psychotropic effects, current psychosis, pervasive developmental disorders, Tourette’s disorder, and neurological disorders. Please note that the participants with current psychiatric conditions (other than psychotic disorders or pervasive developmental disorders) and use of psychotropic medications for psychiatric indications (e.g., stimulants, antipsychotic medications, or selective serotonin reuptake inhibitors [SSRIs]) were not excluded (see Table 1).

A proportion of our participants who were at suicide risk presented with Attention-Deficit/Hyperactivity Disorder (ADHD, *N =* 60), Conduct Disorder (CD, *N =* 48), Major Depressive Disorder (MDD, *N =* 20), Post-Traumatic Stress Disorder (PTSD, *N =* 17), Generalized Anxiety Disorder (GAD, *N =* 31), and Social Anxiety Disorder (SAD, *N =* 27) (see Table 1). Clinical characterization was done through psychiatric interviews by licensed and board-certified child and adolescent psychiatrists with the participants and their parents to adhere closely to common clinical practice.

**Supplementary Section S2:**

*k-NN algorithm:* The k-NN algorithm (function *fitcknn* in MATLAB R2022a) is a non-parametric algorithm that works under the assumption that similar data points (i.e., features) exist in close proximity and, therefore, it considers k number of nearest neighbors (i.e., data points/features) to predict the class for the new (i.e., testing) data points. In other words, classification is made based on feature similarity without any prior knowledge about the data.

*SVM algorithm:* The SVM algorithm (function *fitcsvm* in MATLAB R2022a) identifies a hyperplane (among many possible hyperplanes) in a multi-dimensional space with an objective to distinctly classify the data points (i.e., features). Here, in an N-dimensional space, N is defined as the number of data points/features of interest. The identified hyperplane should have the maximum distance/margin between data points of classes under consideration. Here, maximizing the distance provides more confidence about the accuracy of future/testing data points to be classified in the correct category. Kernels (such as linear, polynomial, Gaussian Radial Basis, or Sigmoid) are provided for the decision functions and were chosen via *fitcsvm* pipeline.

*ENS algorithm:* The ENS algorithm (function *fitcensemble* in MATLAB R2022a) combines several base models to finally generate a single optimal predictive model with a goal of producing the most accurate solution. In the current study, default base models (i.e., ensemble aggregation methods such as Bag, RUSBoost, AdaBoostM1, LogitBoost, and GentleBoost) in *fitcensemble* are used in the ENS algorithm.

**References**

1. Boys Town. Boys Town National Database Manual: Youth Care Data Systems (version 4.5.80.0). Boys Town, NE: Boys Town; 2018.

2. Cull JG, Gill WS. Suicide Probability Scale. West. Psychol. Serv., 1982.
